# Prenatal Exposure to Perfluorocarboxylic Acids (PFCAs) and Fetal and Postnatal Growth in the Taiwan Maternal and Infant Cohort Study

**DOI:** 10.1289/ehp.1509998

**Published:** 2016-02-19

**Authors:** Yan Wang, Margaret Adgent, Pen-Hua Su, Hsiao-Yen Chen, Pau-Chung Chen, Chao A. Hsiung, Shu-Li Wang

**Affiliations:** 1 National Foundation for the Centers for Disease Control and Prevention, Atlanta, Georgia, USA; 2 Epidemiology Branch, National Institute of Environmental Health Sciences, National Institutes of Health, Department of Health and Human Services, Research Triangle Park, North Carolina, USA; 3 Department of Pediatrics, Chung Shan Medical University, Taichung, Taiwan; 4 National Institute of Environmental Health Sciences, National Health Research Institutes, Zhunan, Miaoli County, Taiwan; 5 Institute of Occupational Medicine and Industrial Hygiene, National Taiwan University College of Public Health, Taipei, Taiwan; 6 Institute of Population Health Sciences, National Health Research Institutes, Zhunan, Miaoli County, Taiwan; 7 Department of Public Health, National Defense Medical Center, Taipei, Taiwan; 8 Department of Public Health, College of Public Health, China Medical University, Taichung, Taiwan

## Abstract

**Background:**

Perfluorocarboxylic acids (PFCAs) are environmentally and biologically persistent synthetic chemicals. PFCAs include perfluorooctanoic acid (PFOA; C8) and long-chain PFCAs (C9–C20). Studies examining long-chain PFCAs and fetal and postnatal growth are limited.

**Objectives:**

We investigated the associations of prenatal exposure to long-chain PFCAs with fetal and postnatal growth.

**Methods:**

For 223 Taiwanese mothers and their term infants, we measured PFOA and four long-chain PFCAs (ng/mL) in third-trimester maternal serum; infant weight (kg), length and head circumference (cm) at birth; and childhood weight and height at approximately 2, 5, 8, and 11 years of age. For each sex, we used multivariable linear regression to examine associations between ln-transformed prenatal PFCAs and continuous infant measures, and logistic regression to examine small for gestational age (SGA). Linear mixed models were applied to prenatal PFCAs and childhood weight and height z-scores.

**Results:**

In girls, prenatal perfluorononanoic acid (PFNA), perfluorodecanoic acid (PFDeA), perfluoroundecanoic acid (PFUnDA), and perfluorododecanoic acid (PFDoDA) concentrations were inversely associated with birth weight [e.g., βbirth weight (kg) = –0.06, 95% CI: –0.11, –0.01 per 1 ln-unit PFUnDA increase]; prenatal PFDeA and PFUnDA were associated with elevated odds of SGA; and PFDeA, PFUnDA, and PFDoDA were associated with lower average childhood height z-score. In boys, prenatal PFNA, and PFDoDA were associated with reductions in height at certain ages in childhood, but not with size at birth.

**Conclusions:**

Prenatal exposure to long-chain PFCAs may interfere with fetal and childhood growth in girls, and childhood growth in boys.

**Citation:**

Wang Y, Adgent M, Su PH, Chen HY, Chen PC, Hsiung CA, Wang SL. 2016. Prenatal exposure to perfluorocarboxylic acids (PFCAs) and fetal and postnatal growth in the Taiwan Maternal and Infant Cohort Study. Environ Health Perspect 124:1794–1800; http://dx.doi.org/10.1289/ehp.1509998

## Introduction

Perfluorocarboxylic acids (PFCAs) are a class of synthetic organic chemicals, each consisting of a chain of fluorinated carbons, but varying in chain length (Environment Canada 2012a, 2012b). Specific chemicals include perfluorooctanoic acid (PFOA), containing 8 carbon atoms (C_8_), and long-chain PFCAs, such as perfluorononanoic acid (PFNA; C_9_), perfluorodecanoic acid (PFDeA; C_10_), perfluoroundecanoic acid (PFUnDA; C_11_), and perfluorododecanoic acid (PFDoDA; C_12_). They have been used widely in consumer products such as lubricants, polishes, water-repellant coatings for paper and textiles, fire-fighting foams, and cookware since the 1950s (Post et al. 2012; Prevedouros et al. 2006). PFCAs persist in the environment, and have been widely detected in both wildlife and humans (Lau et al. 2007). Human exposure routes include inhalation and ingestion of PFCAs in dust, water, and food (Fromme et al. 2009).

Leading chemical manufacturers began efforts to reduce PFOA emissions and use around 2000 (Prevedouros et al. 2006). In subsequent years, human serum PFOA concentrations appear to have stabilized (Kato et al. 2011) or declined (Harada et al. 2010; Nøst et al. 2014) in certain geographic regions. Meanwhile, long-chain PFCA levels in human plasma or serum have been increasing in many areas, including the United States (Kato et al. 2011), Sweden (Axmon et al. 2014), Norway (Nøst et al. 2014), and, most notably, countries of East Asia (Harada et al. 2011). Residents of Japan, Korea, and Vietnam had PFNA, PFUnDA, and PFDeA levels comparable with or greater than those of PFOA (Harada et al. 2011). The source of long-chain PFCA exposure may be related to Japanese manufacturing of PFNA, PFUnDA, and other chemicals by fluorotelomer olefin oxidation (Harada et al. 2011; Prevedouros et al. 2006), or related to degradation by-products of other industrial compounds (Lee et al. 2010). In addition, although PFOA has a half-life of approximately 3.5 years in humans (Olsen et al. 2007), long-chain PFCAs have longer half-lives. For example, the median half-life of PFUnDA has been estimated as 7.7 years in men and postmenopausal women; it is approximately 4.4 years in reproductive-age women, for whom menstrual serum clearance is a notable elimination pathway for long-chain, but not short-chain, PFCAs (Zhang et al. 2013).

PFCAs are suspected developmental toxicants. Animal studies suggest PFCAs induce adverse effects on fetal/neonatal mortality and growth (e.g., birth weight and neonatal weight gain), particularly in response to high-dose exposures (Hirata-Koizumi et al. 2012; Kato et al. 2011; Takahashi et al. 2014). In humans, several studies have reported inverse associations between prenatal exposure to PFOA and birth weight (Fei et al. 2007; Lee et al. 2013; Maisonet et al. 2012), but others have not (Hamm et al. 2010; Monroy et al. 2008; Washino et al. 2009). Several recent studies reported null associations between certain long-chain PFCAs, such as PFNA, and birth outcomes (Chen et al. 2012; Jensen et al. 2015; Monroy et al. 2008; Robledo et al. 2015). However, other compounds, such as PFDoDA, have not been studied, nor has the association between many of these compounds and childhood growth. In the present study of a birth cohort in Taiwan, we examined whether serum concentrations of PFOA and four long-chain PFCAs during pregnancy are associated with fetal growth and growth in childhood.

## Methods

### Subjects

The Taiwan Maternal and Infant Cohort Study is a longitudinal cohort study designed to examine prenatal environmental exposures and growth and development in infants and children (Wang et al. 2005). Between December 2000 and November 2001, we invited all pregnant women who visited the local clinics in central Taiwan to participate in the study. We recruited and interviewed 430 women upon their first obstetric visit and collected blood samples during the third trimester. Infants were enrolled at birth and have been followed throughout childhood. In the present study, we included 223 term infants (gestational weeks 37–42, based on the date of mother’s last menstrual period) who had both prenatal PFCA measures and birth outcomes in the present study. Among those, we conducted growth assessments at approximately 2, 5, 8, and 11 years of age. The Human Ethics Committee of the National Health Research Institutes in Taiwan approved the study. Each mother provided signed informed consent for herself and her child before enrollment. Upon reaching elementary school, children also provided informed consent.

### PFCA Exposure Assessment

Prenatal exposure to PFOA and 4 long-chain PFCAs (PFNA, PFDeA, PFUnDA, and PFDoDA) was estimated from third-trimester maternal serum PFCA concentrations. Analytical methods have been described previously (Lien et al. 2011). In brief, the quantification was performed on an Agilent-1200 high performance liquid chromatography system (Agilent, Palo Alto, CA, USA) coupled with a triple-quadrupole mass spectrometer (Sciex API 4000; Applied Biosystems, Foster City, CA, USA). Four calibration standard solutions were analyzed in the same way. The concentrations of specific analytes ranged from 0.25 to 125 ng/mL, with a fixed amount of internal standard (5 ng/mL). By using the standard solutions, we found that the intra-assay coefficients of variation (CVs) for the five analyte concentrations ranged from 0.83 to 7.94% and the interassay CVs were between 1.57 and 24.7%. The limits of quantitation (LOQs), defined as a signal-to-noise ratio of 10, ranged from 0.07 to 0.45 ng/mL.

### Neonatal and Childhood Anthropometric Assessment

Birth weight (kg), length (cm), and head circumference (cm) were measured soon after delivery by nurses in the clinics. Small for gestational age (SGA) was defined as birth weight below the 10th percentile for the gestational age by sex using 1998–2002 Taiwan nationwide singleton birth weight charts (Hsieh et al. 2006). Weight and height were measured by nurses in clinics when infants reached the ages of 2, 5, 8, and 11 years. All birth and childhood weight and height measurements were standardized into *z*-scores by using sex and age-specific Taiwan national references (Chen and Chang 2010).

### Statistical Analysis

We conducted all the analyses among full-term infants, separately by sex. The distributions of prenatal PFOA and long-chain PFCAs were right-skewed, so we used geometric means and percentiles to describe their distributions. For values below the LOQ, we imputed the expected values (E) conditional on being below the LOQ (E[x|x ≤ LOQ]). Specifically, for each PFCA, we estimated mean and variance parameters for a log normal distribution, based on observed percentiles of PFCA concentrations. We then simulated a log normal distribution (*n* = 100,000) using these parameters. From this distribution, we took the mean of all values ≤ LOQ as our imputed value (E[x|x ≤ LOQ]). This method has been shown to attenuate the bias related to exposures under LOQ (Richardson and Ciampi 2003). Prenatal PFCA concentrations were transformed to the natural log (ln) scale in all the following analyses. First, we assessed pair-wise correlations among prenatal PFCAs using Pearson correlation coefficients (*r*). Then we constructed linear regression models to examine each PFCA’s association with birth weight, length, and head circumference; and logistic regression models to assess associations with SGA. To fully assess the exposure, we also modeled prenatal PFCA concentrations as quartiles for continuous birth outcomes (birth weight, length, and head circumference).

We next used mixed linear models to assess childhood weight and height *z*-scores in relation to each prenatal PFCA. An unstructured correlation matrix was selected based on the lowest Akaike’s Information Criterion. We used an interaction term for prenatal PFCA-by-age at follow-up, with age defined categorically as birth and 2, 5, 8, and 11 years, to assess differences in association over time, and to estimate age-specific associations. A statistically significant interaction effect was identified if the interaction term had a *p*-value < 0.10. We also estimated age-specific associations at each time point. In an effort to isolate childhood growth effects from those related to the timing of pubertal onset, we did a sensitivity analysis excluding the age 11 measurements from the linear mixed models.

In all the models, the estimated coefficients (β) or odds ratios (OR) correspond to the unit change in the outcome per 1 ln-unit increase of PFCA concentration (ng/mL). To reduce bias due to potential confounders, we adjusted models for maternal age at delivery (years), education (< high school, high school, part or full college, > college), previous live births (0, ≥ 1), self-reported prepregnancy body mass index (BMI; kg/m^2^), and annual family income (< $20,000 and ≥ $20,000). All analyses were conducted using a complete case approach. We did not adjust for smoking or alcohol consumption because few women smoked (2%) or consumed alcohol during pregnancy (1%). Because maternal weight was not routinely measured, our measurement of maternal weight gain during pregnancy (term weight – prepregnancy weight) was restricted to mothers for whom term weight measures were acquired (*n* = 109). Maternal weight gain and gestational age were additionally included in sensitivity analyses. Finally, we used Student *t*-tests to compare means and chi-square tests to compare frequencies between children who were included in our analysis (*n* = 223) and those who were not (*n* = 207). All the statistical analyses were performed in SAS (SAS Institute Inc., Cary, NC), and all significance levels were set at α = 0.05.

## Results

In the present study, we included 223 full-term infants (106 girls and 117 boys) at birth, and conducted follow-up at ages 2 years (girls: *n* = 80, range = 1.8–2.5 years; boys: *n* = 82, range = 1.8–2.6 years), 5 years (girls: *n* = 50, range = 4.9–5.9; boys: *n* = 51, range = 4.8–6.0), 8 years (girls: *n* = 47, range = 7.8–8.5; boys: *n* = 48, range = 7.8–8.4 years), and 11 years (girls: *n* = 46, range = 10.8–11.4 years; boys: *n* = 48, range = 10.8–11.5 years). Table 1 shows prenatal characteristics, birth outcomes, and childhood weight and height measurements. In this study sample, the mothers averaged 29 years of age at enrollment, and generally did not smoke or consume alcohol during pregnancy. Most of the mothers had at least a high school education, and more than half of the women were primiparous. Forty-nine percent of boys’ mothers and 47% of girls’ mothers reported previous births. The average maternal weight gain during pregnancy was 11.3 ± 5.6 kg and 12.3 ± 5.9 kg for boys’ mothers and girls’ mothers, respectively. There were 18 girls and 17 boys with SGA. Compared with the mothers who enrolled in the study but were not included in our analyses (*n* = 207), mothers who were included (*n* = 223) were more likely to have had a previous birth and, on average, gained less weight during pregnancy (see Table S1). Other characteristics, including prenatal serum PFCA concentrations, did not differ significantly between the groups. All PFCAs were detected in > 70% of the prenatal serum samples (Table 2). Prenatal PFUnDA concentrations had the highest median concentration, followed by PFOA, PFNA, PFDeA, and PFDoDA. The four long-chain PFCAs were highly correlated with each other (*r* ≥ 0.56) but had only low to moderate correlation with PFOA (*r* ≤ 0.34) (see Table S2).

**Table 1:** Characteristics of the pregnant women and children (*n *= 223) in Taiwan Maternal and Infant Cohort Study, 2000–2001.

Characteristics	Female (*n *= 106) [mean ± SD or *n* (%)]	Male (*n *= 117) [mean ± SD or *n* (%)]
Maternal
Age at enrollment (years)
Mean ± SD	29.0 ± 4.6	28.9 ± 4.0
No. of missing	1	1
Prepregnancy BMI (kg/m^2^)
Mean ± SD	20.9 ± 3.2	20.7 ± 3.1
No. of missing	6	5
Weight gain during pregnancy (kg)
Mean ± SD	11.3 ± 5.6	12.3 ± 5.9
No. of missing	60	64
Previous live birth ≥ 1 [*n* (%)]	52 (49)	55 (47)
Education [*n* (%)]
< High school	7 (7)	4 (3)
High school	46 (43)	48 (42)
Part or full college	34 (32)	44 (38)
> College	19 (18)	19 (17)
No. of missing	1	1
Annual family income (US$) [*n* (%)]
< 20,000	40 (38)	48 (42)
≥ 20,000	65 (62)	66 (58)
No. of missing	1	3
Smoking during pregnancy [*n* (%)]	2 (2)	1 (1)
Drinking alcohol during pregnancy [*n* (%)]	1 (1)	4 (3)
Children
Gestational weeks at birth [median (range)]	39 (37–42)	39 (37–42)
Birth weight (kg) (mean ± SD)	3.0 ± 0.4	3.2 ± 0.4
Birth length (cm) (mean ± SD)	51.0 ± 2.2	51.9 ± 2.2
Birth head circumference (cm) (mean ± SD)	33.2 ± 1.4	33.7 ± 1.3
SGA [*n* (%)]	18 (18)	17 (15)
Weight (kg) (mean ± SD)
2 years	12.6 ± 1.7	13.5 ± 2.0
5 years	19.9 ± 3.7	20.6 ± 3.5
8 years	27.9 ± 6.1	29.5 ± 6.3
11 years	40.2 ± 10.1	44.3 ± 11.0
Height (cm) (mean ± SD)
2 years	87.3 ± 3.1	88.8 ± 4.3
5 years	110.1 ± 4.2	111.0 ± 5.2
8 years	129.8 ± 4.9	130.0 ± 5.8
11 years	147.9 ± 6.8	146.8 ± 7.0
Weight *z*-score (mean ± SD)
At birth	–0.5 ± 1.1	–0.1 ± 0.7
2 years	0.1 ± 1.3	0.5 ± 1.3
5 years	0.3 ± 1.8	0.8 ± 1.8
8 years	0.6 ± 2.2	1.2 ± 2.2
11 years	0.3 ± 2.0	1.7 ± 2.0
Height *z*-score (mean ± SD)
At birth	1.2 ± 1.4	1.2 ± 1.4
2 years	–0.8 ± 1.0	–0.7 ± 1.3
5 years	–0.4 ± 1.0	–0.2 ± 1.0
8 years	0.5 ± 1.1	0.5 ± 1.2
11 years	0.3 ± 1.1	0.6 ± 1.2

**Table 2:** Prenatal PFOA and long-chain PFCAs concentrations in serum samples in the Taiwan Maternal and Infant Cohort Study, 2000–2001.

Prenatal exposure (ng/mL)	Molecular formula	Detection rate (%)	Geometric mean (95% CI)	Min^*a*^	25th	50th	75th	90th	Max
Female (*n *= 106)
PFOA	C_7_F_15_CO_2_H	86	1.98 (1.69, 2.32)	0.39	1.57	2.34	3.43	4.64	7.70
PFNA	C_8_F_17_CO_2_H	97	1.44 (1.19, 1.74)	0.08	0.84	1.58	2.42	4.54	10.30
PFDeA	C_9_F_19_CO_2_H	75	0.37 (0.32, 0.42)	0.16	0.16	0.43	0.64	0.83	1.24
PFUnDA	C_10_F_21_CO_2_H	90	2.89 (2.12, 3.94)	0.10	1.46	3.31	9.15	14.05	38.00
PFDoDA	C_11_F_23_CO_2_H	82	0.30 (0.25, 0.35)	0.06	0.23	0.37	0.51	0.67	1.26
Male (*n *= 117)
PFOA	C_7_F_15_CO_2_H	85	1.97 (1.67, 2.31)	0.39	1.35	2.37	3.47	4.60	13.50
PFNA	C_8_F_17_CO_2_H	97	1.39 (1.16, 1.66)	0.08	0.92	1.55	2.60	4.24	10.25
PFDeA	C_9_F_19_CO_2_H	73	0.40 (0.35, 0.46)	0.16	0.16	0.46	0.72	0.96	1.57
PFUnDA	C_10_F_21_CO_2_H	92	3.12 (2.36, 4.14)	0.10	1.67	3.52	9.35	15.40	43.10
PFDoDA	C_11_F_23_CO_2_H	80	0.28 (0.23, 0.33)	0.06	0.19	0.37	0.56	0.73	1.17
Abbreviations: Max, maximum; Min, minimum. 25th, 50th, 75th, and 90th are percentiles. ^***a***^Minimum values correspond to imputed values for concentrations < LOQ. Values were imputed as expected values (E) conditional on being below the LOQ (E[x|x ≤ LOQ]).									

Table 3 shows βs [95% confidence intervals (CIs)] from linear regression models for associations of prenatal PFCAs with birth outcomes. We observed that prenatal PFNA, PFDeA, PFUnDA, and PFDoDA concentrations were significantly associated with birth weight among girl infants, with adjusted β values of –0.08 (–0.16, 0.00), –0.14 (–0.26, –0.02), –0.06 (–0.11, –0.01), and –0.12 (–0.21, –0.02), respectively. These estimates suggest that, for example, there is a 0.08-kg decrease in birth weight in response to a 1–ln-unit increase in prenatal PFNA concentration (ng/mL), or a 0.008-kg decrease in birth weight in response to 10% increase in prenatal PFNA. We also observed significant associations between PFDoDA and head circumference (adjusted β = –0.38; –0.74, –0.02) as well as between PFDeA and PFUnDA and the odds of SGA among girls, with ORs (95% CIs) of 3.14 (1.07, 9.19) and 1.83 (1.01, 3.32), respectively. In boys, most of the associations between prenatal PFCAs and birth outcomes were close to the null and all were nonsignificant. Additional adjustment for gestational age at delivery or maternal weight gain during pregnancy did not substantially change our results (data not shown). Results were mostly similar when prenatal PFCAs were modeled as quartiles. Consistent with the linear models, the highest quartiles of PFNA, PFDeA, and PFUnDA concentration in girls were associated with significant decreases in birth weight, compared with the lowest quartile (see Table S3; boys’ data not shown).

**Table 3:** β (95% CIs) or OR (95% CIs) for associations of prenatal PFOA and long-chain PFCAs with birth outcomes by infant sex in the Taiwan Maternal and Infant Cohort Study, 2000–2001.

Prenatal exposure	Birth weight β (95% CI)^*a*^	Birth length β (95% CI)^*a*^	Birth head circumference β (95% CI)^*a*^	SGA OR (95% CI)^*a*^
Female
PFOA	–0.08 (–0.18, 0.01)	–0.32 (–0.92, 0.28)	0.11 (–0.26, 0.47)	1.48 (0.63, 3.48)
PFNA	–0.08 (–0.16, 0.00)*	–0.38 (–0.88, 0.13)	–0.28 (–0.59, 0.02)	2.03 (0.90, 4.56)
PFDeA	–0.14 (–0.26, –0.02)*	–0.47 (–1.23, 0.30)	–0.37 (–0.85, 0.10)	3.14 (1.07, 9.19)*
PFUnDA	–0.06 (–0.11, –0.01)*	–0.31 (–0.62, 0.00)	–0.14 (–0.34, 0.05)	1.83 (1.01, 3.32)*
PFDoDA	–0.12 (–0.21, –0.02)*	–0.39 (–0.98, 0.21)	–0.38 (–0.74, –0.02)*	1.98 (0.73, 5.33)
Male
PFOA	0.04 (–0.05, 0.12)	0.31 (–0.22, 0.84)	0.06 (–0.24, 0.36)	0.63 (0.32, 1.13)
PFNA	0.02 (–0.06, 0.11)	–0.17 (–0.67, 0.33)	–0.10 (–0.39, 0.19)	0.96 (0.52, 1.76)
PFDeA	0.04 (–0.06, 0.15)	–0.06 (–0.68, 0.57)	–0.12 (–0.48, 0.24)	0.71 (0.33, 1.52)
PFUnDA	0.01 (–0.04, 0.06)	–0.06 (–0.37, 0.25)	–0.06 (–0.24, 0.12)	0.87 (0.61, 1.25)
PFDoDA	0.04 (–0.04, 0.12)	0.05 (–0.46, 0.55)	–0.05 (–0.35, 0.24)	0.78 (0.43, 1.42)
^***a***^Models were adjusted for family annual income, maternal age at delivery, maternal education, maternal previous live children, and maternal prepregnancy BMI. *p < 0.05.				

**Figure 1:**
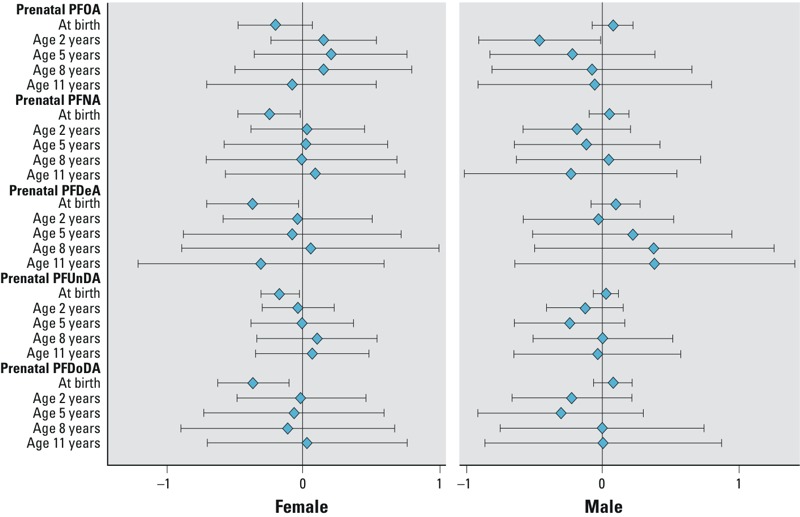
Age-specific β (95% CIs) for associations of prenatal PFOA and long-chain PFCAs with children’s weight *z*-score PFCA (per 1–ln-unit increase) by sex at age 0–11 years in the Taiwan Maternal and Infant Cohort Study, 2000–2001. Estimates are β (95% CIs) from linear regression models, which were adjusted for family annual income, maternal age upon delivery, maternal education, maternal previous live children, and maternal prepregnancy BMI.

**Figure 2:**
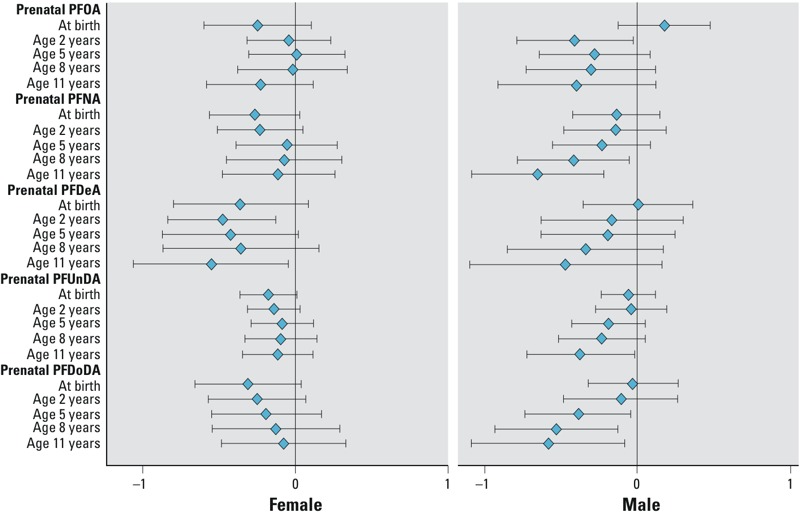
Age-specific β (95% CIs) for associations of prenatal PFOA and long-chain PFCAs with children’s height *z*-score (per 1–ln-unit increase) by sex at age 0–11 years in the Taiwan Maternal and Infant Cohort Study, 2000–2001. Estimates are β (95% CIs) from linear regression models, which were adjusted for family annual income, maternal age upon delivery, maternal education, maternal previous live children, and maternal prepregnancy BMI.

Table 4 shows βs (95% CIs) from mixed linear models. In girls, all prenatal PFCAs show negative trends of associations with average weight and height *z*-scores over childhood. Prenatal PFDeA, PFUnDA, and PFDoDA were significantly associated with lower average weight *z*-score over the childhood with adjusted β values of –0.32 (–0.63, 0.00), –0.15 (–0.28, –0.02), and –0.30 (–0.55, –0.06), respectively. No significant interactions between prenatal PFCAs and age at follow-up were found (*p* > 0.10); however, age-specific estimates suggest that reductions in girls’ weight *z*-score were limited to the time of birth and null at later ages (Figure 1). We also observed significant reductions in girls’ average childhood height *z*-score in association with prenatal PFDeA, PFUnDA, and PFDoDA concentrations, with adjusted β of –0.52 (–0.80, –0.24), –0.14 (–0.27, –0.01), and –0.25 (–0.49, 0.00), respectively. Interaction terms for prenatal PFCA concentration-by-age were not significant (*p* > 0.10). For each PFCA, the magnitude of association was generally consistent across childhood (Figure 2). In a sensitivity analysis restricting the analysis to 8 years of age or younger in girls, we observed similar results for the overall associations (data not shown).

**Table 4:** β (95% CIs) for associations of PFOA and long-chain PFCAs with weight *z*-score and height *z*-score on average during childhood by children’s sex in the Taiwan Maternal and Infant Cohort Study, 2000–2001.

Prenatal exposure	Weight *z*-score^*a*^	Height *z*-score^*a*^
Female
PFOA	–0.14 (–0.39, 0.11)	–0.15 (–0.38, 0.08)
PFNA	–0.20 (–0.42, 0.01)	–0.21 (–0.42, 0.00)
PFDeA	–0.32 (–0.63, 0.00)*	–0.52 (–0.80, –0.24)**
PFUnDA	–0.15 (–0.28, –0.02)*	–0.14 (–0.27, –0.01)*
PFDoDA	–0.30 (–0.55, –0.06)*	–0.25 (–0.49, 0.00)*
Male
PFOA	0.03 (–0.11, 0.18)	0.01 (–0.24, 0.25)
PFNA	0.01 (–0.13, 0.15)	–0.15 (–0.37, 0.08)^*b*^
PFDeA	0.09 (–0.09, 0.26)	–0.05 (–0.34, 0.23)
PFUnDA	0.01 (–0.07, 0.10)	–0.08 (–0.22, 0.06)
PFDoDA	0.06 (–0.08, 0.20)	–0.17 (–0.40, 0.06)^*b*^
^***a***^Values listed were β (95% CIs) from mixed linear regression models, which were adjusted for family annual income, maternal age upon delivery, maternal education, maternal previous live children, and maternal prepregnancy BMI. ^***b***^Significant interactions between prenatal PFCAs and child age were found for these models; estimates do not adequately characterize the associations. *p < 0.05. **p < 0.01.		

In boys, the average associations between prenatal PFCAs and weight *z*-scores across childhood were close to the null (Table 4). No significant interactions between prenatal PFCAs and age were observed (*p* > 0.10). For height *z*-scores, however, we observed significant interactions with follow-up age for prenatal PFNA (*p* = 0.02) and PFDoDA exposure (*p* = 0.07). In general, age-specific estimates became increasingly negative with increasing age for all PFCAs (Figure 2). These estimates were significant for prenatal PFNA and boys’ height *z*-score at ages 8 and 11 years; and prenatal PFDoDA and boys’ height *z*-score at ages 5, 8, and 11 years.

## Discussion

In the present study, we found that prenatal exposures to long-chain PFCAs were inversely associated with girls’ size at birth and with boys’ and girls’ height across childhood. Specifically, in girls, we observed associations between prenatal PFNA, PFDeA, PFUnDA, and PFDoDA and decreased birth weight; prenatal PFDeA and PFUnDA and increased odds of being SGA; and prenatal PFDoDA and smaller head circumference at birth. Associations with girls’ weight did not appear to extend past birth into childhood, but average height *z*-scores were significantly reduced in association with prenatal PFDeA, PFUnDA, and PFDoDA concentrations. We observed a different pattern of association for boys, in which birth outcomes were not associated with prenatal PFCA exposure, but childhood height was reduced in association with increasing exposure. For prenatal PFNA and PFDoDA, these inverse associations with height were significant only at later ages of follow-up. In addition, because we used separate models to estimate associations with each compound due to high correlations among the exposures, we cannot attribute any of the observed associations to any single PFCA.

In contrast to PFOA, which has been widely studied with respect to birth outcomes, epidemiologic studies of long-chain PFCAs are sparse in the current literature. In a Canadian birth cohort, several long-chain compounds, including PFNA and PFDeA, were assessed during pregnancy (*n* = 101) and delivery (*n* = 105) (Monroy et al. 2008). In this study, PFDeA was detected at too low a frequency to be further studied; neither PFNA nor PFOA was found to be associated with birth weight. Similar results were shown in a Taiwanese birth cohort where notably high concentrations of PFUnDA in cord blood (*n* = 429; geometric mean, 10.26 ng/mL) were inversely, but not significantly, associated with birth weight, length, and head circumference; PFNA was associated with an increase in birth length and a decrease in ponderal index (Chen et al. 2012). Last, a study in the United States (*n* = 230) observed no association between preconception maternal PFOA, PFNA, or PFDeA and birth size (Robledo et al. 2015).

Despite strong geographic and temporal similarities in study samples, our study and the study by Chen et al. (2012) arrived at inconsistent conclusions regarding long-chain PFCAs and birth outcomes. The reasons for inconsistencies may be attributable to methodological differences related to study design and analysis. First, a notable difference exists in the studies’ exposure assessment methods (i.e., cord blood vs. maternal serum). Although cord blood PFCA concentrations correlate with the maternal concentrations (Gützkow et al. 2012; Monroy et al. 2008), it is likely that long-chain PFCAs have different efficiencies in crossing the placenta (Gützkow et al. 2012). For example, detection frequencies for certain PFCAs, such as PFNA, are lower in cord blood than maternal serum (Monroy et al. 2008), consistent with a 68% detection frequency in cord blood in the study by Chen et al. (2012) and a 96% detection frequency in maternal serum in the present study. Therefore, maternal serum and cord blood measures are likely capturing different exposures within the maternal–fetal system, and these distinct exposures may be acting on fetal growth through different mechanisms. Next, our birth outcome findings were most prominently observed in girls, whereas sex-specific estimates were not reported by Chen et al. (2012) We do not have a clear explanation for sex-specific growth effects, but experimental and observational evidence show sex-specific endocrine effects that may be related to growth and development. For example, PFDoDA exposure has been shown to disrupt estrogen biosynthesis and estrogen signal transduction in pubertal female rats (Shi et al. 2009), and PFUnDA and PFOA have been associated with decreased follicle-stimulating hormone and sex hormone–binding globulin, respectively, in adolescent girls (Tsai et al. 2015). It has also been postulated that male and female fetuses adapt differently to maternal stress, with females, but not males, exhibiting reduced growth in response to maternal inflammation (Clifton 2010). However, to our knowledge, such an effect has not been investigated in relation to PFCAs.

In addition to birth outcomes, our study also observed an inverse association between long-chain PFCAs and average childhood height among girls, and height among boys at later ages of follow-up. We also observed a significant inverse association between prenatal long-chain PFCAs and girls’ average childhood weight, but this was attributed mainly to associations at birth. To our knowledge, no epidemiologic studies to date have assessed childhood growth in relation to prenatal exposure to long-chain PFCAs. However, a recent experimental animal study found that exposure to PFNA during gestation reduced pups’ postnatal weight in a dose-dependent manner (Das et al. 2015). It is possible that the observed associations in our study population reflect prenatal effects of PFCAs on postnatal growth. However, we cannot exclude postnatal PFCA exposures as possible contributors to these associations, such as exposures occurring through breast milk or the childhood environment. Additionally, for both boys and girls, age-specific associations for weight and height *z*-scores were imprecisely estimated due to loss to follow-up over time, and thus should be interpreted cautiously.

Compared with Western populations (Axmon et al. 2014; Kato et al. 2011; Nøst et al. 2014), mothers in our study were exposed to comparatively high levels of long-chain PFCAs, particularly PFUnDA, whereas exposure to PFOA was relatively low. This exposure profile is consistent with other Asian populations (Harada et al. 2011), and may be of concern due to suspected increased toxicity of long-chain PFCAs relative to PFOA. For example, C_9_, C_10_, and C_11_ PFCAs are more hydrophobic and are less readily excreted through urine than C_8_ compounds (Kudo et al. 2001). Long-chain PFCAs concentrate in bile, and undergo enterohepatic recirculation, potentially contributing to or prolonging the toxicity of long-chain PFCAs (Goecke-Flora and Reo 1996). In our study, associations tended to be strongest among the long-chain PFCA compounds. Although many of the observed associations were small (e.g., girls’ average reductions in height correspond to a one-half or less of a standard deviation decrease in height per ln-unit increase in exposure), they may represent meaningful shifts in growth across a highly exposed population. Additionally, we observed associations between certain PFCAs and birth weight that are similar in magnitude to other established intrauterine growth risk factors. For example, prenatal smoking is associated with an approximate 0.2-kg reduction in birth weight (Butler et al. 1972; Ellard et al. 1996); similarly, the highest quartile of prenatal PFUnDA exposure, compared with the lowest, was associated with a 0.3-kg reduction in birth weight among girls. Further, in our previous study on the same population, we reported that four long-chain PFCAs were inversely associated with both maternal and fetal thyroid hormones (Wang et al. 2014). Together, these findings suggest that long-chain PFCAs may be more of a public health concern in Taiwan than PFOA or other short-chain perfluorinated substances.

The greatest strength of our study is its longitudinal design, in that we were able to assess both birth outcomes and childhood growth in relation to a well-measured prenatal exposure to long-chain PFCAs, which has not been well characterized in other studies. We were also able to control for potentially confounding prenatal factors, such as prepregnancy BMI, previous births, and markers of socioeconomic status. However, some limitations should be noted. First, we did not account for blood volume expansion during pregnancy. Blood volume typically increases between the second and third trimesters of pregnancy (Hytten 1985). This expansion serves to improve utero–placental blood flow for nutrient transfer to the fetus (Rosso et al. 1992). Reduced maternal blood volume is associated with fetal growth restriction (Salas and Rosso 1998), whereas increases in volume may contribute to decreasing PFOA concentrations across the trimesters of pregnancy (Fei et al. 2007; Monroy et al. 2008). Therefore, our observed associations between third-trimester PFCA concentrations and reduced birth weight may plausibly be biased due to confounding by maternal blood volume. Additional limitations include our relatively small sample size which inhibited our ability to evaluate other clinical birth outcomes, such as low birth weight or preterm birth. Finally, we lack information on lactational exposure to long-chain PFCAs during infancy or other postnatal exposures in the childhood, which may also affect childhood growth.

## Conclusion

Our results suggest that prenatal exposure to long-chain PFCAs may interfere with girls’ weight at birth, as well as boys’ and girls’ height in childhood.

## Supplemental Material

(113 KB) PDF
