# Supplementary material for: Prenatal Exposure to Perfluorocarboxylic Acids (PFCAs) and Fetal and Postnatal Growth in the Taiwan Maternal and Infant Cohort Study
Source: Environ Health Perspect. 2016 Feb 19;124(11):1794–800. doi: 10.1289/ehp.1509998 (PMC5089898; doi:10.1289/ehp.1509998)
Supplement: (113 KB) PDF [file ehp.1509998.s001.acco.pdf]

Environ Health Perspect
DOI: 10.1289/ehp.1509998

Note to readers with disabilities: *EHP* strives to ensure that all journal content is accessible to all readers. However, some figures and Supplemental Material published in *EHP* articles may not conform to 508 standards due to the complexity of the information being presented. If you need assistance accessing journal content, please contact ehp508@niehs.nih.gov. Our staff will work with you to assess and meet your accessibility needs within 3 working days.

Supplemental Material

Prenatal Exposure to Perfluorocarboxylic Acids (PFCAs) and Fetal and Postnatal Growth in the Taiwan Maternal and Infant Cohort Study

Yan Wang, Margaret Adgent, Pen-Hua Su, Hsiao-Yen Chen, Pau-Chung Chen, Chao A. Hsiung, and Shu-Li Wang

Table of Contents

Table S1. Prenatal Characteristic Comparisons between Children Included in the Present Study and Not Included in the Taiwan Maternal and Infant Cohort Study

Table S2. Pair-wise Person Correlation Coefficients among Ln-transformed PFCAs in the Taiwan Maternal and Infant Cohort Study (n=223)

Table S3. β (95% CIs) for Associations of Prenatal PFOA and Long-chain PFCAs with Birth Outcomes in Female Infants in the Taiwan Maternal and Infant Cohort Study, 2000-2001

Table S1. Prenatal Characteristic Comparisons between Children Included in the Present Study and Not Included in the Taiwan Maternal and Infant Cohort Study

Characteristics	Children included (n=223)	Children not included (n=207)	p value[a]
**Mothers**			
Age at enrollment (years), Mean ± SD	29.1 ± 4.2	28.2 ± 4.3	0.06
Pre-pregnancy BMI (kg/m^2), Mean ± SD	20.8 ± 3.0	20.5 ± 3.2	0.36
Weight gain during pregnancy (kg)[b], Mean ± SD	11.6 ± 5.9	13.4 ± 5.9	0.002
PFOA (µg/mL), GM (95% CI)	2.0 (1.8, 2.2)	1.9 (1.6, 2.2)	0.6
PFNA (µg/mL), GM (95% CI)	1.4 (1.2, 1.6)	1.4 (1.1, 1. 6)	0.7
PFDeA (µg/mL), GM (95% CI)	0.4 (0.3, 0.4)	0.4 (0.3, 0.5)	0.4
PFUnDA (µg/mL), GM (95% CI)	3.0 (2.4, 3.7)	3.4 (2.7, 4.4)	0.4
PFDoDA (µg/mL), GM (95% CI)	0.3 (0.2, 0.3)	0.3 (0.3, 0.4)	0.3
Education, n(%)			
< High school	9 (5)	15 (7)	0.9
High school	83 (42)	96 (43)	
Part college	74 (37)	81 (36)	
≥ college	34 (17)	32 (14)	
Previous live birth ≥ 1, n(%)	100 (50)	82 (36)	0.005
Annual family income (US $), n(%)			
< 20, 000	80 (40)	103 (47)	0.2
≥ 20, 000	118 (60)	118 (53)	
Smoking during pregnancy, n(%)	3 (1.5)	5 (2.3)	0.61
Drinking alcohol during pregnancy, n(%)	0 (0)	2 (0.9)	
**Infants**			
Sex, n(%)			
Female	98 (49)	73 (48)	0.9
Male	104 (51)	79 (52)	

a. p values were obtained from student *t* tests or chi-square tests.

b. N for weight gain during pregnancy was 109 children included and 72 not included.

Table S2. Pair-wise Person Correlation Coefficients among Ln-transformed PFCAs in the Taiwan Maternal and Infant Cohort Study (n=223)

	PFOA	PFNA	PFDeA	PFUnDA	PFDoDA
PFOA	1	0.32**	0.34**	0.13	0.14*
PFNA		1	0.62**	0.81**	0.80**
PFDeA			1	0.56**	0.57**
PFUnDA				1	0.86**
PFDoDA					1

* $p<0.05$; ** $p<0.001$.

Table S3. β (95% CIs) for Associations of Prenatal PFOA and Long-chain PFCAs with Birth Outcomes[a] in Female Infants in the Taiwan Maternal and Infant Cohort Study, 2000-2001

Prenatal PFCAs (ng/mL)	Birth weight[b]	Birth length[b]	Birth head circumference[b]
PFOA			
≤ 1.57 (Reference)			
> 1.57-2.34	-0.12 (-0.35, 0.12)	-0.39 (-1.80, 1.02)	0.08 (-0.81, 0.96)
> 2.34-3.43	-0.05 (-0.27, 0.18)	0.78 (-0.57, 2.13)	-0.01 (-0.85, 0.84)
> 3.43	-0.11 (-0.34, 0.12)	-0.60 (-1.98, 0.77)	0.49 (-0.38, 1.35)
PFNA			
≤ 0.84 (Reference)			
> 0.84-1.58	-0.15 (-0.38, 0.08)	-0.18 (-1.62, 1.27)	-1.25 (-2.10, -0.39)**
> 1.58-2.42	-0.25 (-0.48, -0.03)*	-0.64 (-2.04, 0.76)	-1.07 (-1.90, -0.24)*
> 2.42	-0.26 (-0.49, -0.03)*	-0.87 (-2.28, 0.55)	-0.76 (-1.59, 0.08)
PFDeA			
≤ 0.16 (Reference)			
> 0.16-0.43	0.00 (-0.22, 0.23)	0.07 (-1.33, 1.48)	0.05 (-0.83, 0.93)
> 0.43-0.64	-0.12 (-0.33, 0.10)	-0.20 (-1.56, 1.16)	-0.30 (-1.15, 0.55)
> 0.64	-0.23 (-0.44, -0.01)*	-0.75 (-2.09, 0.59)	-0.67 (-1.51, 0.16)
PFUnDA			
≤ 1.46 (Reference)			
> 1.46-3.31	-0.15 (-0.37, 0.06)	-0.94 (-2.30, 0.42)	-0.47 (-1.30, 0.37)
> 3.31-9.15	-0.19 (-0.41, 0.03)	-0.56 (-1.93, 0.81)	-0.50 (-1.35, 0.34)
> 9.15	-0.36 (-0.58, -0.14)**	-1.65 (-3.04, -0.26)*	-0.67 (-1.52, 0.19)
PFDoDA			
≤ 0.23 (Reference)			
> 0.23-0.37	-0.00 (-0.23, 0.23)	-0.37 (-1.80, 1.06)	-0.49 (-1.38, 0.40)
> 0.37-0.51	-0.19 (-0.41, 0.03)	-0.85 (-2.21, 0.51)	-0.27 (-1.12, 0.58)
> 0.51	-0.18 (-0.40, 0.05)	-1.01 (-2.40, 0.38)	-0.52 (-1.38, 0.35)

*p<0.05; **p<0.01.

a. Prenatal PFCAs by quartiles were not fit for SGA because of the small sample size of SGA.

b. Models were adjusted for family annual income, maternal age at delivery, maternal education, maternal previous live children, and maternal pre-pregnancy BMI.
